# Intermuscular coherence as an early biomarker for amyotrophic lateral sclerosis: The protocol for a prospective, multicenter study

**DOI:** 10.1371/journal.pone.0303053

**Published:** 2024-05-22

**Authors:** Naoum P. Issa, Serdar Aydin, Eric Polley, Nathan Carberry, Mark A. Garret, Sean Smith, Ali A. Habib, Nicholas W. Baumgartner, Betty Soliven, Kourosh Rezania

**Affiliations:** 1 University of Chicago Medical Center, Chicago, IL, United States of America; 2 University of Miami, Coral Gables, FL, United States of America; 3 Massachusetts General Hospital, Boston, MA, United States of America; 4 Washington University, St. Louis, MO, United States of America; 5 University of California, Irvine, Irvine, CA, United States of America; 6 Rush University Medical Center, Chicago, IL, United States of America; Public Library of Science, UNITED KINGDOM

## Abstract

**Objective:**

To describe the protocol of a prospective study to test the validity of intermuscular coherence (IMC) as a diagnostic tool and biomarker of upper motor neuron degeneration in amyotrophic lateral sclerosis (ALS).

**Methods:**

This is a multicenter, prospective study. IMC of muscle pairs in the upper and lower limbs is gathered in ∼650 subjects across three groups using surface electrodes and conventional electromyography (EMG) machines. The following subjects will be tested: 1) neurotypical controls; 2) patients with symptomatology suggestive for early ALS but not meeting probable or definite ALS by Awaji Criteria; 3) patients with a known ALS mimic. The recruitment period is between 3/31/2021 and 12/31/2025. Written consent will be sought from the subject or the subject’s legally authorized representative during enrollment.

**Results:**

The endpoints of this study include: 1) whether adding IMC to the Awaji ALS criteria improve its sensitivity in early ALS and can allow for diagnosis earlier; 2) constructing a database of IMC across different ages, genders, and ethnicities.

**Significance:**

This study may validate a new inexpensive, painless, and widely available tool for the diagnosis of ALS.

## Introduction

Amyotrophic lateral sclerosis (ALS) is a progressive, uniformly fatal neurodegenerative disorder characterized by the loss of motor neurons in the brain and spinal cord. The loss of motor neurons results in paralysis and death, typically within 5 years and with 60% mortality by 3 years [1, 2]. ALS is characterized by significant phenotypic, genetic, and pathogenic heterogeneity. Phenotypic variability arises in the extent and location of upper motor neuron (UMN) or lower motor neuron (LMN) involvement, with typical ALS patients having a combination of both UMN and LMN symptomatology [1, 3].

On average, there is a delay of 12–16 months from the onset of symptoms to the diagnosis of ALS [4–6]. Currently, diagnosis of ALS is based on a history of progressive weakness, exam findings of LMN and UMN dysfunction, and exclusion of diseases that mimic ALS [7, 8]. One common reason for diagnostic uncertainty and delay in diagnosis is the lack of UMN features on physical exam, as UMN signs are not always present in early ALS [9, 10]. Needle EMG features of acute and chronic denervation were set equivalent to physical exam findings of LMN dysfunction in Awaji criteria to improve the diagnostic sensitivity [8]. While needle EMG is a reliable and widely available tool to confirm LMN degeneration, physical exam is the only practical way to assess UMN loss at the present time. An objective, widely available, electrophysiological assessment of UMN dysfunction is missing from current ALS diagnostic criteria.

Intermuscular coherence (IMC) has been proposed as a marker of UMN dysfunction and preliminary reports suggest it can differentiate patients with motor neuron disease from neurotypical subjects [11–13]. The output from the motor cortex is in part oscillatory, so a portion of the activity in separate muscles involved in a motor task oscillates at the same frequency [14, 15]. As a result, the cortical signal to muscles can be assessed by measuring the coherence between two co-activated muscles (IMC) [14–21]. IMC is a method for imputing the extent of central (UMN) drive to muscles and is calculated from the patterns of surface EMG activity in muscle pairs, which is independent of EMG assessments of LMN dysfunction like interference pattern and motor unit action potential sorting and analysis [20, 22]. We here outline the protocol for Intermuscular Coherence as a Biomarker for ALS (ALS-IMC) (NCT05104710), a prospective study to validate IMC as a diagnostic tool for assessing UMN dysfunction in ALS.

Several considerations affected the development of the protocol. First, the diagnostic dilemma with ALS is usually not, whether an abnormality exists, rather whether the etiology of the abnormality is ALS or a disorder that mimics ALS. To evaluate whether IMC can distinguish between subjects with ALS and an ALS-mimic requires multiple comparator groups, including patients with ALS and patients with a variety of ALS-mimics. This is particularly useful in attempting to differentiate patients with ALS from those with lower-motor neuron mimics. Second, because IMC profiles vary based on muscles assessed, the task performed during measurement, and potentially by subject demographics, a distribution of test-specific IMC profiles must be generated in neurotypical subjects. Finally, the ideal diagnostic test would reduce the time to a definitive diagnosis, so the protocol was designed to assess whether IMC could provide a diagnosis at an earlier time point than is currently the typical.

## Materials and methods

### Study outline

The study is organized into two sections. The first establishes the distribution of test-specific IMC profiles in neurotypical subjects across demographic groups (Neurotypical IMC distributions). The second (IMC in ALS and ALS mimics) asks 1) whether adding abnormal IMC values to the Awaji criteria results in ALS diagnosis earlier in the course of disease, and 2) whether IMC is a valid tool to distinguish ALS from different ALS mimics. Written consent will be sought from the subject or the subject’s legally authorized representative during enrollment.

### Study subjects

In Aim 1, IMC distributions will be assessed in neurotypical subjects who report no current neurological issues. Subjects will be recruited to sample demographic variables including age, sex, race, and ethnicity. Measurements will be made in subjects from each decade between 20 and 80 years old, for male or female sex, African-American or White race, or Hispanic or non-Hispanic ethnicity. Depending on the overlap in ethnic and racial distributions, we anticipate that data from 240 subjects will be needed to sample the demographic space and determine how IMC varies across the neurotypical population. A measurement of IMC in both arms and both legs will be made.

In Aim 2, IMC profiles will be collected from 410 subjects (N selected based on power analysis described below) who present with symptoms suggestive for ALS but not meeting probable or definitive ALS classification using Awaji Criteria, i.e. patients with uncategorized or possible ALS according to the Awaji Criteria. Patients diagnosed with various mimic disorders will also be included. The specific inclusion and exclusion criteria are as follows:

**Inclusion:** a) Patients who present to one of the sites for upper or lower limb weakness, spastic gait, muscle wasting and/or fasciculations, dysphagia, dysarthria, shortness of breath, hyperreflexia or pathological reflexes, or findings of muscle denervation in previous EMG studies. b) Patients with a LMN mimic disease such as spinal muscular atrophy, spinal-bulbar muscular atrophy (Kennedy’s disease), multifocal motor neuropathy and other motor predominant neuropathy or radiculopathy, inclusion body myositis, other myopathies and neuromuscular junction disorders. c) Patients with an UMN mimic disease, such as primary lateral sclerosis (PLS), hereditary spastic paraparesis, ischemic or demyelinating disease with purely motor symptomatology.

**Exclusion:** a) Patients who are classified as probable or definite ALS by Awaji criteria prior to initial study evaluation, b) significant sensory loss in the weak or spastic limbs, c) significant musculoskeletal or neuropathic pain, d) inability or unwillingness to provide informed consent, e) inability to perform the study-related task, and f) subjects who have taken a GABA agonist (e.g. baclofen or benzodiazepines) within 24 hours of the study procedure.

To characterize disease state at the time of testing, information will be collected through chart review and subject interviews, including: a) date and site of onset of symptoms, b) height and weight, c) diagnostic category based on Awaji criteria, c) topographical location and pattern of UMN and LMN symptoms at the time of the IMC test.

### Measurement of IMC

IMC will be calculated from surface EMG measurements of co-activated muscle pairs. The muscle pair tested in the upper extremity consists of the biceps and brachioradialis [11]; two muscle pairs will be evaluated in the lower limbs: tibialis anterior (TA) and extensor digitorum brevis (EDB), and gastrocnemius lateralis (GL) and gastrocnemius medialis (GM). When testing the upper extremity, the subject performs an antigravity “hold” task in which the forearm is held parallel to the ground and the upper arm is perpendicular to the ground. For assessment of the TA and EDB pair, the subject dorsiflexes the ankle and extends the toes at the same time. For assessment of the GL/GM pair, the subject will be seated with the foot flat on the floor then the heel will be elevated. Each position will be held for 30 seconds at a time, with three repetitions separated by ∼30-second rest blocks.

Surface EMG will be recorded using a sample electrode over the muscle belly and a reference electrode. In preliminary experiments the reference electrode was placed 3 cm from the sample electrode; in ongoing experiments this reference position is being compared to a reference placed over a distant bony prominence. Waveforms will be recorded using clinical EMG systems, either Natus (http://neuro.natus.com/) or Cadwell (http://cadwell.com/), which are available at all the research centers. Signals will be sampled digitally at a minimum of 1000 Hz with pre-amplification low-pass filtering at ∼1/3 the sampling frequency to avoid aliasing.

IMC from the paired EMG signals will be calculated over a range of frequencies, using the algorithm previously reported [23]. Scalar metrics will be calculated by averaging IMC between 10 and 30 Hz (IMC-αβ) or 20 and 40 Hz (IMC-βγ). The precision of a coherence measurement is determined by the number of segments used in the calculation. The confidence limit is defined as:

$$CL=1-\alpha^{1/(S-1)}$$

in which α is the desired significance level (0.05), *S* is the number of segments analyzed, and values below the CL are not statistically different from zero (no coherence) [24].

### Determination of IMC normal-abnormal threshold

**Fig 1:**
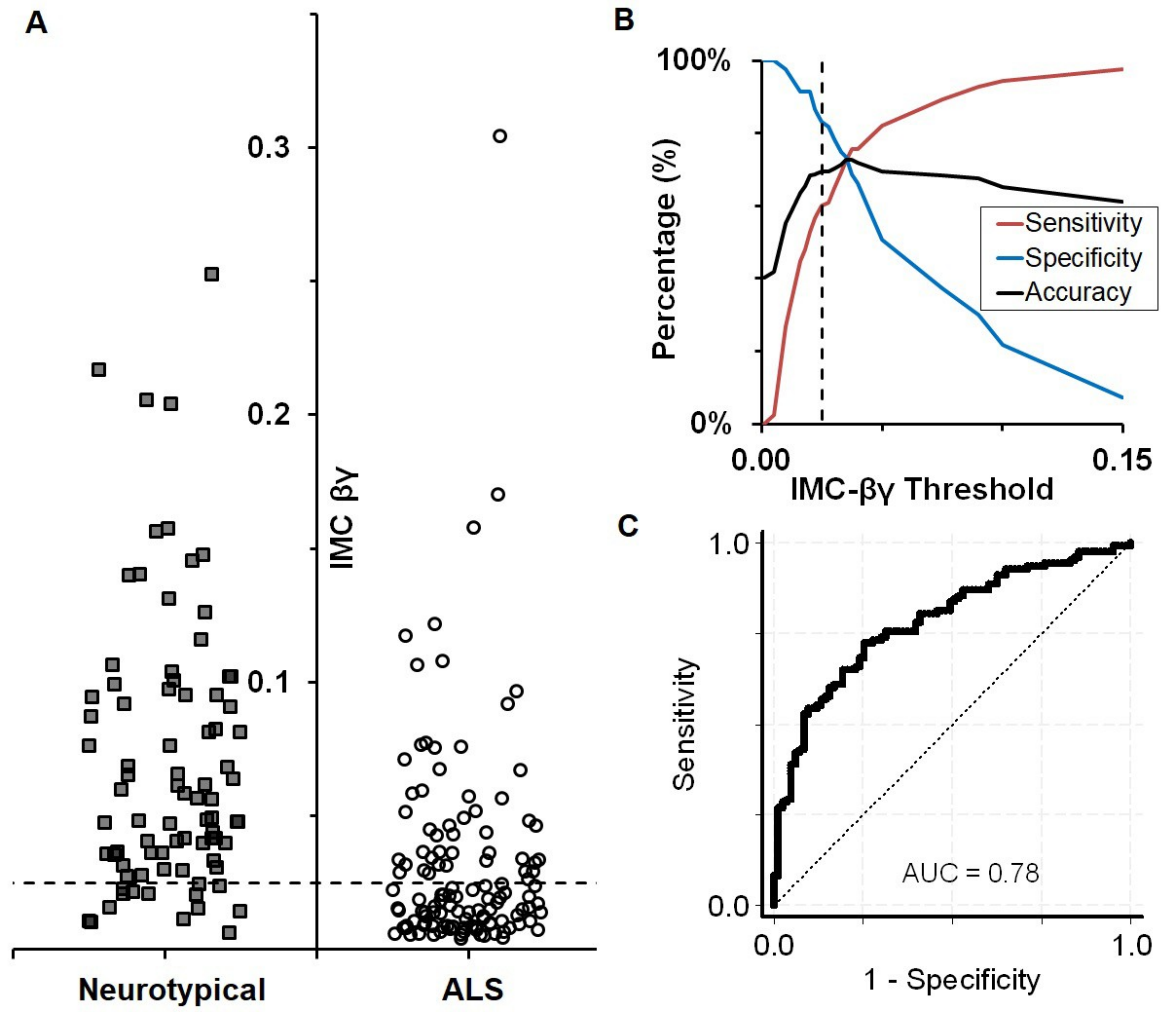
Threshold determination of IMC-βγ. A. IMC-βγ values in neurotypical subjects (n = 83) and ALS patients (n = 123). The dashed line is the cutoff threshold of 0.025, below which IMC-βγ is considered abnormal. B. Accuracy, sensitivity and specificity of IMC-βγ in distinguishing neurotypical from ALS patients, as a function of the IMC value used as a cutoff for abnormal. Dashed line shows the cutoff displayed in A. C. ROC curve showing sensitivity and 1-specificity of the IMC test as a function IMC cutoff value. Area under the curve 0.78.

Because the ALS diagnostic criteria require a binary determination of the presence or absence of UMN dysfunction, we will set a threshold IMC value below which UMN function is considered abnormal. In a preliminary study we measured IMC-βγ in the right arm of 83 neurotypical controls and 123 subjects with an established ALS diagnosis. These preliminary data were collected prior to initiation of the currently described study protocol, under a separately reviewed and approved IRB protocol (University of Chicago IRB15-0237). IMC measurements were made with the same procedure described above for acquiring surface EMG recordings from the arm. Based on the data shown in Fig 1A (S1 Table; these data have not been previously reported) we found that IMC cutoff values between 0.02 and 0.035 had equivalent diagnostic accuracy using a bipolar recording configuration in the arm (Fig 1B). There was an expected reciprocity between sensitivity and specificity at different cutoff values (Fig 1B). Since the study assesses IMC-βγ added to the current Awaji Criteria, it may increase the sensitivity of the criteria, but runs a risk of reducing the specificity. To minimize the risk to specificity, the cutoff value was selected to optimize accuracy and specificity at the expense of some sensitivity. A cutoff value of 0.025 was selected and will be used in the arm study. A separate cutoff value will be determined for the leg based on the exploratory study on these regions. With this cutoff value, arm IMC-βγ, independent of other diagnostic criteria, has a sensitivity of 60% and a specificity of 83% for ALS compared to neurotypical controls. A low arm IMC-βγ gave an odds ratio (OR) of 7.3 for having ALS (95% CI 3.7 to 14.5; p<0.0001), with an area under the receiver operating characteristic curve of 0.78.

Two preliminary studies will be carried out to confirm the threshold levels. First, measurements of threshold will be made for different reference electrode positions for the arm and leg in the same subjects. The location of the reference electrodes affects the dynamic range of the coherence measurement and might affect the optimal threshold. Characterizing the effect of reference location on dynamic range and cutoff will also provide information on the reproducibility of the test if reference locations vary in clinical practice. Second, an IMC threshold will be identified for IMC measured in the legs.

### Comparison of Awaji classification to Awaji+IMC classification

**Fig 2:**
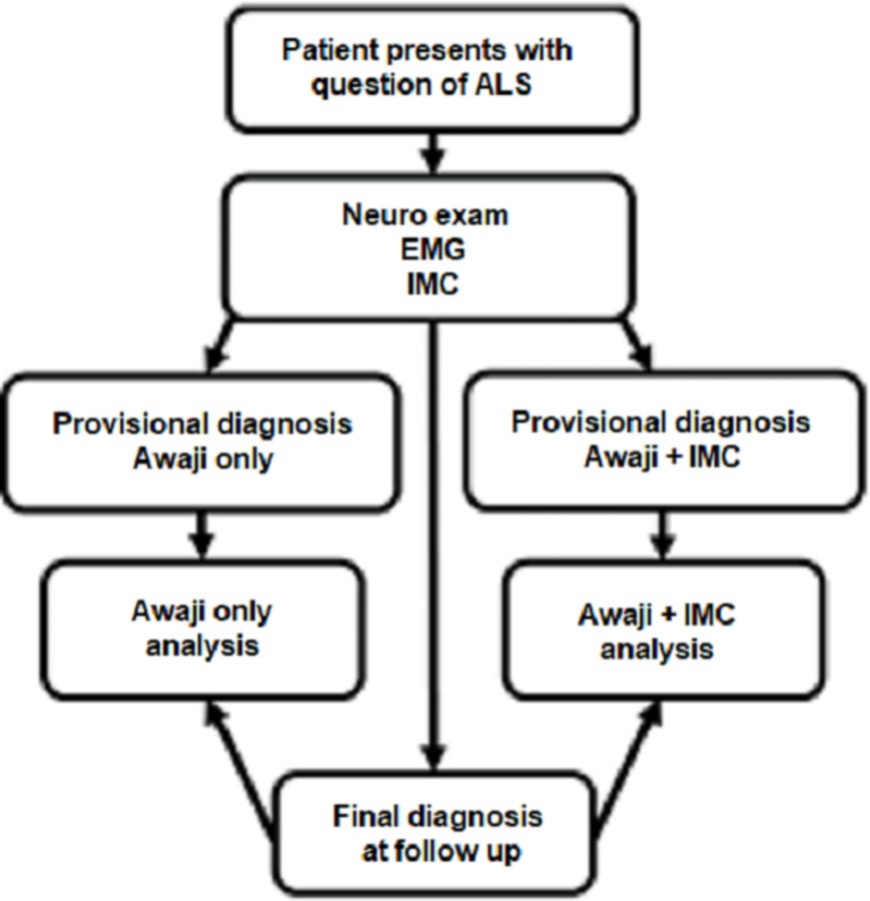
Experimental flow of Aim 2. Two provisional diagnoses will be made at initial presentation: one based on the Awaji criteria alone, and one based on the Awaji criteria with IMC assessment. Every 6 months a patient’s clinical record will be reviewed to assign a final diagnosis. After a final diagnosis is determined, the performance at initial presentation of the Awaji+IMC criteria will be compared to that of Awaji alone.

To determine whether incorporating an IMC metric would improve diagnostic certainty of ALS, in Aim 2 we will prospectively compare the performance of two diagnostic schemes applied at initial presentation (Fig 2). Classification according to the Awaji Criteria will serve as the baseline diagnosis. The second diagnostic scheme will consist of the Awaji Criteria plus IMC, in which an abnormal IMC value will be considered the equivalent of an upper motor neuron sign in that limb. At six-month intervals each case will be assessed to determine if a definitive diagnosis has been made, and at what delay after symptom onset the diagnosis was made. The null hypothesis is that there is no difference in delay to an ALS diagnosis by incorporating IMC measurements into the Awaji Criteria.

### Assessment of final diagnosis

A diagnosis of ALS or non-ALS condition will be made by the treating team and reviewed by a standing committee of neuromuscular specialists. Disagreement in diagnosis will be resolved by consensus; if a consensus cannot be reached the subject will be excluded from analysis. The clinical review committee will be blind to the IMC results and provisional diagnoses.

### Statistical analysis

**Fig 3:**
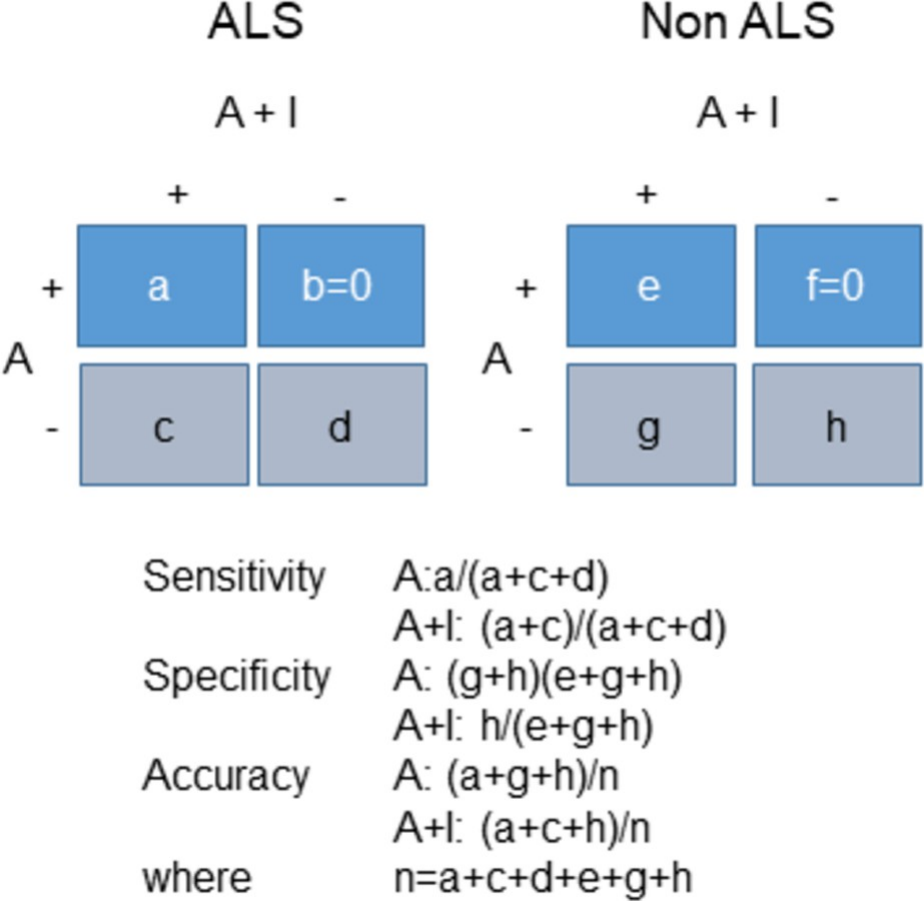
Cross-classification of initial Awaji with Awaji+IMC results by final diagnosis. A = Awaji criteria at initial visit, A+I = Awaji+IMC criteria at initial visit, +: definite or probable category, -: possible or uncategorized.

To determine whether adding IMC changes diagnostic accuracy, sensitivity, or specificity at initial assessment we will estimate and compare the sensitivity, specificity and overall accuracy of the Awaji+IMC criteria at initial evaluation relative to the Awaji criteria alone. If Awaji+IMC criteria detect more cases of ALS at initial evaluation, the distribution of diagnostic certainty will shift to higher certainty. A probable or definite category of Awaji criteria or a clinical diagnosis of ALS given by an ALS expert (as per *Assessment of final diagnosis*) will constitute a positive test result. The data will be summarized in two 2x2 tables as depicted in Fig 3. While there is not an independent “gold standard” for the diagnosis of ALS, very few patients (∼6%) in probable or definite ALS categories in the Awaji criteria turn out not to have ALS (denoted “e” in Fig 3) [25]. Given the role of Awaji Criteria measured in follow up appointments as a determinant of diagnosis, the specificity of the assessments at first visit will always be high. In addition, by construct if the Awaji criteria are positive then Awaji+IMC will be positive and thus the frequencies b and f in Fig 2 are structural zeros. In essence the question being addressed is the extent to which the sensitivity is increased by the incorporation of IMC relative to the decrease in specificity; these quantities will be estimated by $\Delta \hat{sens}=c/(a+c+d)$ and $\Delta \hat{spec}=-g/(e+g+h)$, respectively. The difference in accuracy will be estimated by *(c − g)/n*.

To determine whether adding IMC results in an earlier diagnosis of ALS, the time to diagnosis will be determined by when a patient achieves a diagnosis of ALS (as per *Assessment of final diagnosis*). At the end of the study, the time from first noticed symptom to diagnosis will be compared between Awaji and Awaji+IMC criteria using a one-tailed paired T-test.

### Sample size justification

The sample size of the study was selected to determine whether IMC can materially reduce the number of false negatives (i.e., improve sensitivity) associated with the Awaji Criteria. The initial visit Awaji criteria have a sensitivity of 56–61% (probable or definite at initial visit with eventual diagnosis of ALS) [25, 26]. We will therefore target a true change in sensitivity (*Δsens*) of 9% and will test against the null hypothesis of *Δsens* of 4% (insufficient improvement). Power analysis suggests a total of 243 cases with a final diagnosis of ALS will be needed for an α < 0.05 and a power of > 90%. To estimate the number of patients that need to be screened we assume the distribution of patients enrolled is similar to that reported by Geevasinga et al [26] in which ∼60% of patients with “possible ALS” or “uncategorized” by the Awaji criteria at first visit were eventually diagnosed with ALS, and ∼40% were diagnosed with an ALS mimic. Therefore, the total sample size required is 243/0.6 = 405. We will enroll 410 patients, which will provide ∼160 patients for estimating the change in specificity.

To determine the magnitude of diagnostic time reduction that could be detected with this sample size, a power analysis was performed. We assume a possible reduction in time-to-diagnosis of 0 to 5 months, with a uniform distribution and standard deviation of 1.4 months. An N of 243 cases of ALS would provide 90% power, at α = 0.05, to detect a true reduction in time-to-diagnosis of 0.3 months.

### Study organization

This is a multi-center study carried out in the ALS clinics at the University of Chicago (UC, primary site), Washington University, Massachusetts General Hospital, University of California at Irvine, and University of Miami. The protocol was approved by the Institutional Review Board at the University of Chicago (The University of Chicago Biological Sciences Division/University of Chicago Medical Center, FWA00005565) with local review at the participating institutions (University of California, Irvine, University of Miami, Washington University, Massachusetts General Hospital). The recruitment period is between 3/31/2021 and 12/31/2025. Both aims have the same recruitment period. All raw data will be transferred to and analyzed at the primary site (UC) to assure consistency in analysis and application of diagnostic criteria. Data from evaluations of individual patients will be uploaded by participating sites to a secure cloud-based data repository (RedCap and OwnCloud), including de-identified patient characteristics, exam and EMG reports, and raw sEMG data. A physician at the testing center will make a provisional classification according to the Awaji Criteria at the time of initial testing, IMC calculations will be performed by a technician who is blinded to the provisional classification, and provisional classification according to the Awaji+IMC criteria will be made after IMC calculation. Determination of a final diagnosis will be made as per *Assessment of final diagnosis* (Fig 2). Statistical analyses will be performed by a biostatistician who is not involved in determining the clinical diagnoses or the IMC calculation. Follow up clinical data will be abstracted by a research assistant from the electronic health records into a RedCap database.

## Discussion

A paucity of disease-specific biomarkers to identify ALS patients early and objectively track disease progression has contributed to the slow development and testing of disease modifying compounds [27]. This is compounded by a need for high diagnostic certainty to enter ALS trials, as patients who are “uncategorized” by the Awaji criteria or are in the “possible” category are generally ineligible to participate in clinical trials. Earlier diagnosis should, therefore, be a priority for improving outcomes in ALS. One common reason for diagnostic uncertainty is the lack of UMN symptomatology on physical exam. There is accumulating evidence on the use of IMC as a biomarker for motor neuron diseases [11, 12, 28].

This study seeks to validate a simple technique for improving the diagnoses of ALS using IMC derived from surface recordings of muscle activity. The method is inexpensive, non-invasive, and could be implemented widely as it is performed using standard EMG equipment. Interpretation of IMC measurements would be performed with a standalone software package that analyzes surface EMG data and calculates IMC values, providing a binary qualitative interpretation of the results as normal or abnormal. IMC analysis can be done in patients who have significant muscle weakness as a Medical Research Council strength of ≥3/5 in the tested muscles will be adequate to perform the assessment.

A previous study by Jaiser et al suggested that IMC in the β frequency range in muscle pairs in the upper and lower limbs is not affected by age or sex [29]. As our study tasks are different from those employed in the aforementioned study, we will build a database of IMC across different ages, genders and ethnicities, to provide demographic-specific cutoff values for normal vs abnormal values of IMC. We will also investigate if IMC better differentiates ALS from LMN or UMN predominant mimics. If IMC is a good measure of UMN dysfunction, then it should not by itself distinguish ALS from UMN disorders like PLS or hereditary spastic paraplegia. Conversely, IMC should better differentiate between ALS and LMN-predominant mimics than the Awaji criteria alone.

## Conclusion

This study protocol is designed to test the validity of IMC as a diagnostic tool in ALS. If successfully validated, an abnormal IMC result could lead to earlier ALS diagnoses, increasing the number of patients with early ALS who will be eligible for available and oncoming treatments.

## Supporting information

S1 Table Preliminary data table. IMC-βγ values measured in preliminary experiments and used to determine the IMC-βγ threshold between neurotypical and ALS subjects are presented. These data are plotted in Fig 1. Age at exam is specified in years. ID: subject identifier, n/a: not available.
(DOCX)
