# Supplementary material for: Intermuscular coherence as an early biomarker for amyotrophic lateral sclerosis: The protocol for a prospective, multicenter study
Source: PLoS One. 2024 May 22;19(5):e0303053. doi: 10.1371/journal.pone.0303053 (PMC11111088; doi:10.1371/journal.pone.0303053)
Supplement: S1 Table — IMC-βγ values measured in preliminary experiments and used to determine the IMC-βγ threshold between neurotypical and ALS subjects are presented. These data are plotted in Fig 1. Age at exam is specified in years. ID: subject identifier, n/a: not available. (DOCX) [file pone.0303053.s001.docx]

| **ID** | **Final Diagnosis** | **Sex** | **Age at Exam** | **IMC-βγ Amplitude** |
| --- | --- | --- | --- | --- |
| N001 | Neurotypical | Female | 62 | 0.040340 |
| N002 | Neurotypical | Female | 52 | 0.140014 |
| N003 | Neurotypical | Female | 73 | 0.090592 |
| N004 | Neurotypical | Female | 60 | 0.046701 |
| N005 | Neurotypical | Female | 48 | 0.035415 |
| N006 | Neurotypical | Female | 50 | 0.101750 |
| N007 | Neurotypical | Female | 34 | 0.252452 |
| N008 | Neurotypical | Female | 31 | 0.061134 |
| N009 | Neurotypical | Female | 65 | 0.030169 |
| N010 | Neurotypical | Female | 23 | 0.039588 |
| N011 | Neurotypical | Female | 66 | 0.027026 |
| N012 | Neurotypical | Female | 22 | 0.101659 |
| N013 | Neurotypical | Female | 32 | 0.147410 |
| N014 | Neurotypical | Female | 25 | 0.022374 |
| N015 | Neurotypical | Female | 46 | 0.027531 |
| N016 | Neurotypical | Female | 69 | 0.014963 |
| N017 | Neurotypical | Female | 56 | 0.005912 |
| N018 | Neurotypical | Female | 67 | 0.094911 |
| N019 | Neurotypical | Female | n/a | 0.075996 |
| N020 | Neurotypical | Female | 41 | 0.047435 |
| N021 | Neurotypical | Female | 29 | 0.098997 |
| N022 | Neurotypical | Female | 27 | 0.130767 |
| N023 | Neurotypical | Female | 23 | 0.057955 |
| N024 | Neurotypical | Female | 30 | 0.139596 |
| N025 | Neurotypical | Female | 39 | 0.216705 |
| N026 | Neurotypical | Female | 61 | 0.059662 |
| N027 | Neurotypical | Female | n/a | 0.096958 |
| N028 | Neurotypical | Female | 32 | 0.064930 |
| N029 | Neurotypical | Female | 40 | 0.048167 |
| N030 | Neurotypical | Female | 56 | 0.041184 |
| N031 | Neurotypical | Female | 39 | 0.094218 |
| N032 | Neurotypical | Female | 67 | 0.041273 |
| N033 | Neurotypical | Female | 60 | 0.075762 |
| N034 | Neurotypical | Female | 61 | 0.157264 |
| N035 | Neurotypical | Female | 56 | 0.068313 |
| N036 | Neurotypical | Male | 76 | 0.039604 |
| N037 | Neurotypical | Male | 53 | 0.080967 |
| N038 | Neurotypical | Male | 56 | 0.047159 |
| N039 | Neurotypical | Male | 50 | 0.145059 |
| N040 | Neurotypical | Male | 34 | 0.067690 |
| N041 | Neurotypical | Male | 35 | 0.115716 |
| N042 | Neurotypical | Male | 27 | 0.205219 |
| N043 | Neurotypical | Male | 30 | 0.036020 |
| N044 | Neurotypical | Male | 67 | 0.021354 |
| N045 | Neurotypical | Male | 63 | 0.013942 |
| N046 | Neurotypical | Male | 53 | 0.010273 |
| N047 | Neurotypical | Male | 71 | 0.080904 |
| N048 | Neurotypical | Male | 44 | 0.029091 |
| N049 | Neurotypical | Male | 32 | 0.100163 |
| N050 | Neurotypical | Male | 24 | 0.023254 |
| N051 | Neurotypical | Male | 24 | 0.015243 |
| N052 | Neurotypical | Male | 30 | 0.055999 |
| N053 | Neurotypical | Male | 53 | 0.063489 |
| N054 | Neurotypical | Male | 68 | 0.035945 |
| N055 | Neurotypical | Male | 68 | 0.035930 |
| N056 | Neurotypical | Male | 69 | 0.032967 |
| N057 | Neurotypical | Male | 34 | 0.029435 |
| N058 | Neurotypical | Male | 77 | 0.060833 |
| N059 | Neurotypical | Male | 70 | 0.009834 |
| N060 | Neurotypical | Male | 50 | 0.094965 |
| N061 | Neurotypical | Male | 37 | 0.091726 |
| N062 | Neurotypical | Male | 32 | 0.056375 |
| N063 | Neurotypical | Male | 29 | 0.156034 |
| N064 | Neurotypical | Male | 33 | 0.034884 |
| N065 | Neurotypical | Male | 46 | 0.082101 |
| N066 | Neurotypical | Male | 77 | 0.041106 |
| N067 | Neurotypical | Male | 68 | 0.043548 |
| N068 | Neurotypical | Male | 43 | 0.024033 |
| N069 | Neurotypical | Male | 62 | 0.040278 |
| N070 | Neurotypical | Male | 79 | 0.030970 |
| N071 | Neurotypical | Male | 70 | 0.125703 |
| N072 | Neurotypical | Male | 65 | 0.086728 |
| N073 | Neurotypical | Male | 38 | 0.010977 |
| N074 | Neurotypical | Male | 64 | 0.020633 |
| N075 | Neurotypical | Male | 59 | 0.203963 |
| N076 | Neurotypical | Male | 52 | 0.047600 |
| N077 | Neurotypical | Male | 66 | 0.020103 |
| N078 | Neurotypical | Male | 68 | 0.106248 |
| N079 | Neurotypical | Male | 41 | 0.020328 |
| N080 | Neurotypical | Male | 66 | 0.047920 |
| N081 | Neurotypical | Male | 31 | 0.048880 |
| N082 | Neurotypical | Male | 66 | 0.065275 |
| N083 | Neurotypical | Male | 27 | 0.103685 |
| A001 | ALS | Male | 65 | 0.005601 |
| A002 | ALS | Male | 71 | 0.023546 |
| A003 | ALS | Female | 70 | 0.013386 |
| A004 | ALS | Male | 61 | 0.022000 |
| A005 | ALS | Female | 64 | 0.033825 |
| A006 | ALS | Female | 53 | 0.304156 |
| A007 | ALS | Female | 31 | 0.106017 |
| A008 | ALS | Male | 77 | 0.029083 |
| A009 | ALS | Female | 68 | 0.070896 |
| A010 | ALS | Female | 78 | 0.021814 |
| A011 | ALS | Female | 64 | 0.014966 |
| A012 | ALS | Male | 59 | 0.006142 |
| A013 | ALS | Female | 74 | 0.019697 |
| A014 | ALS | Female | 68 | 0.024000 |
| A015 | ALS | Female | 71 | 0.014193 |
| A016 | ALS | Male | 55 | 0.033779 |
| A017 | ALS | Male | 43 | 0.046117 |
| A018 | ALS | Female | 67 | 0.008325 |
| A019 | ALS | Female | 79 | 0.014448 |
| A020 | ALS | Male | 79 | 0.005179 |
| A021 | ALS | Female | 51 | 0.008012 |
| A022 | ALS | Male | 66 | 0.033175 |
| A023 | ALS | Male | 74 | 0.007980 |
| A024 | ALS | Male | 64 | 0.013836 |
| A025 | ALS | Male | 70 | 0.009539 |
| A026 | ALS | Male | 67 | 0.003599 |
| A027 | ALS | Female | 60 | 0.016897 |
| A028 | ALS | Male | 54 | 0.005658 |
| A029 | ALS | Male | 67 | 0.007874 |
| A030 | ALS | Male | 78 | 0.005316 |
| A031 | ALS | Male | 45 | 0.051391 |
| A032 | ALS | Male | 71 | 0.008927 |
| A033 | ALS | Female | 46 | 0.045843 |
| A034 | ALS | Female | 66 | 0.031594 |
| A035 | ALS | Female | 58 | 0.012255 |
| A036 | ALS | Female | 71 | 0.008433 |
| A037 | ALS | Female | 49 | 0.015433 |
| A038 | ALS | Male | 69 | 0.005123 |
| A039 | ALS | Male | 64 | 0.020156 |
| A040 | ALS | Female | 61 | 0.020889 |
| A041 | ALS | Female | 55 | 0.015683 |
| A042 | ALS | Female | 67 | 0.007890 |
| A043 | ALS | Female | 67 | 0.012485 |
| A044 | ALS | Female | 57 | 0.013601 |
| A045 | ALS | Female | 55 | 0.004971 |
| A046 | ALS | Male | 51 | 0.013599 |
| A047 | ALS | Female | 65 | 0.012187 |
| A048 | ALS | Female | 57 | 0.004107 |
| A049 | ALS | Male | 63 | 0.006434 |
| A050 | ALS | Male | 70 | 0.051197 |
| A051 | ALS | Male | 64 | 0.056204 |
| A052 | ALS | Male | 74 | 0.014748 |
| A053 | ALS | Male | 61 | 0.010265 |
| A054 | ALS | Female | 56 | 0.043356 |
| A055 | ALS | Female | 67 | 0.017990 |
| A056 | ALS | Male | 74 | 0.008016 |
| A057 | ALS | Male | 51 | 0.005486 |
| A058 | ALS | Female | 53 | 0.010289 |
| A059 | ALS | Female | 73 | 0.007061 |
| A060 | ALS | Female | 67 | 0.157351 |
| A061 | ALS | Male | 62 | 0.008129 |
| A062 | ALS | Male | 48 | 0.096371 |
| A063 | ALS | Male | 45 | 0.007376 |
| A064 | ALS | Male | 47 | 0.017222 |
| A065 | ALS | Male | 67 | 0.008432 |
| A066 | ALS | Female | 55 | 0.006394 |
| A067 | ALS | Female | 59 | 0.011427 |
| A068 | ALS | Female | 77 | 0.016158 |
| A069 | ALS | Female | 41 | 0.036323 |
| A070 | ALS | Female | 61 | 0.117237 |
| A071 | ALS | Male | 26 | 0.014611 |
| A072 | ALS | Male | 52 | 0.056823 |
| A073 | ALS | Male | 79 | 0.008591 |
| A074 | ALS | Male | 65 | 0.007707 |
| A075 | ALS | Male | 64 | 0.010139 |
| A076 | ALS | Male | 69 | 0.005291 |
| A077 | ALS | Female | 44 | 0.035843 |
| A078 | ALS | Female | 71 | 0.007894 |
| A079 | ALS | Male | 74 | 0.009225 |
| A080 | ALS | Female | 74 | 0.022489 |
| A081 | ALS | Female | 66 | 0.169739 |
| A082 | ALS | Female | 72 | 0.075141 |
| A083 | ALS | Female | 72 | 0.044391 |
| A084 | ALS | Female | 64 | 0.009571 |
| A085 | ALS | Male | 62 | 0.013097 |
| A086 | ALS | Female | 64 | 0.107538 |
| A087 | ALS | Male | 54 | 0.013863 |
| A088 | ALS | Female | 69 | 0.048965 |
| A089 | ALS | Female | 66 | 0.028842 |
| A090 | ALS | Male | 45 | 0.018806 |
| A091 | ALS | Female | 66 | 0.019002 |
| A092 | ALS | Male | 60 | 0.042852 |
| A093 | ALS | Male | 57 | 0.028885 |
| A094 | ALS | Female | 58 | 0.009207 |
| A095 | ALS | Male | 57 | 0.066789 |
| A096 | ALS | Female | 57 | 0.058206 |
| A097 | ALS | Female | 71 | 0.023408 |
| A098 | ALS | Male | 77 | 0.059012 |
| A099 | ALS | Male | 51 | 0.018316 |
| A100 | ALS | Male | 72 | 0.032228 |
| A101 | ALS | Male | 62 | 0.076283 |
| A102 | ALS | Female | 66 | 0.076949 |
| A103 | ALS | Female | 52 | 0.030938 |
| A104 | ALS | Female | 69 | 0.035974 |
| A105 | ALS | Female | 67 | 0.028470 |
| A106 | ALS | Male | 51 | 0.025862 |
| A107 | ALS | Male | 59 | 0.013583 |
| A108 | ALS | Male | 65 | 0.020156 |
| A109 | ALS | Male | 72 | 0.011058 |
| A110 | ALS | Female | 61 | 0.033191 |
| A111 | ALS | Female | 64 | 0.091589 |
| A112 | ALS | Female | 54 | 0.067025 |
| A113 | ALS | Male | 64 | 0.042195 |
| A114 | ALS | Male | 67 | 0.036137 |
| A115 | ALS | Female | 55 | 0.019482 |
| A116 | ALS | Male | 80 | 0.032983 |
| A117 | ALS | Male | 59 | 0.007024 |
| A118 | ALS | Male | 61 | 0.031318 |
| A119 | ALS | Female | 77 | 0.121275 |
| A120 | ALS | Male | 68 | 0.047814 |
| A121 | ALS | Female | 75 | 0.019545 |
| A122 | ALS | Male | 55 | 0.075552 |
| A123 | ALS | Male | 69 | 0.028145 |
|  |  |  |  |  |

n/a = not available
